# Selection on embryonic haemoglobin in an elevational generalist songbird

**DOI:** 10.1098/rsbl.2022.0105

**Published:** 2022-10-12

**Authors:** Elizabeth J. Beckman, Walter Vargas Campos, Phred M. Benham, C. Jonathan Schmitt, Zachary A. Cheviron, Christopher C. Witt

**Affiliations:** ^1^ Museum of Southwestern Biology and Department of Biology, University of New Mexico, Albuquerque, NM 87131, USA; ^2^ Museum of Vertebrate Zoology and Department of Integrative Biology, University of California, Berkeley, CA 94720, USA; ^3^ Centro de Ornitología y Biodiversidad, Calle Sta. Rita 105, Oficina 202, Santiago de Surco, Lima, Perú; ^4^ Department of Organismic and Evolutionary Biology and Museum of Comparative Zoology, Harvard University, Cambridge, MA 02138, USA; ^5^ Division of Biological Sciences, University of Montana, Missoula, MT, USA

**Keywords:** adaptation, high elevation, hypoxia, haemoglobin, development, birds

## Abstract

Animals developing at high elevation experience a suite of environmental challenges, most notably the low partial pressure of oxygen (*P*O_2_) in ambient air. In low *P*O_2_, bird species with high-elevation ancestry consistently demonstrate higher hatching success than lowland counterparts, suggesting highland birds are adapted to restricted O_2_ (hypoxia) in early development. Haemoglobin (Hb), the critical oxygen-transport protein, is a likely target of *P*O_2_-related selection across ontogeny since Hb isoforms expressed at distinct developmental stages demonstrate different O_2_ affinities. To test if Hb function is under *P*O_2_-related selection at different ontogenetic stages, we sampled a songbird, the hooded siskin (*Spinus magellanicus*), across two approximately 4000 m elevational transects. We sequenced all of the loci that encode avian Hb isoforms, and tested for signatures of spatially varying selection by comparing divergence patterns in Hb loci to other loci sampled across the genome. We found strong signatures of diversifying selection at non-synonymous sites in loci that contribute to embryonic (*α*^π^, *β*^H^) and definitive (*β*^A^) Hb isoforms. This is the first evidence for selection on embryonic haemoglobin in high-elevation Neoaves. We conclude that selection on Hb function at brief, but critical stages of ontogeny may be a vital component to high elevation adaptation in birds.

## 1. Introduction

Selection pressures over the course of a lifetime determine whole-organism fitness. Animals developing at high elevation must surmount a number of environmental challenges. In particular, the decrease in the partial pressure of oxygen (*P*O_2_) with increasing elevation is a chronic stressor for metabolically active animals [1]. Consequently, many high-elevation lineages have evolved genetic adaptations to cope with this environmental challenge [2]. The stress associated with low *P*O_2_ (hypoxia) may be extreme at specific ontogenetic stages given the different energetic costs of growth and reproduction [3]. Further, the path of oxygen from the air to respiring tissues changes fundamentally from prenatal to postnatal life in many vertebrates including birds and mammals [4,5].

At the earliest life stages, birds breeding at high elevation are vulnerable to unique environmental stresses. Low barometric pressure impacts gas diffusion across the eggshell; consequently, water loss increases and the oxygen gradient between the environment and the egg is reduced [6,7]. Hatching success at low *P*O_2_ is significantly reduced in birds with lowland ancestry [4,8,9]. However, high-elevation birds demonstrate embryonic growth rates, incubation times and hatching success *in situ* similar to lowland counterparts [6,9–11]. These differences may reflect evolved strategies to tolerate low barometric pressure during development. Compared to lowland relatives, embryos in high-elevation populations exhibit increased eggshell resistance to minimize water loss [6,12], enhanced oxygen delivery via the critical oxygen-transport protein haemoglobin (Hb) [13] and distinct transcript abundance profiles [14].

Little is known about the genetic basis of adaptations that improve embryonic survival at high elevation. However, haemoglobin, a tetramer comprised subunits from the *α*-globin and *β*-globin gene families, is a likely target of *P*O_2_-related selection across ontogeny. First, Hb isoforms with different O_2_ affinities are expressed at different life stages [4]. Second, in the first week of incubation, embryonic Hb facilitates oxygen transport when diffusion is most restricted across the eggshell [7]. Third, birds at high elevation frequently demonstrate a stronger intrinsic Hb–O_2_ binding affinity than lowland relatives due to spatially varying selection on the globins *α*^D^, *α*^A^ and *β*^A^ which contribute to definitive Hb isoforms after the first week of incubation [14–17]. Last, in *Anas* ducks, the embryonic Hb locus *β^ε^*, along with *β*^A^, is strongly differentiated at several non-synonymous sites between high and low populations, suggesting that embryonic Hb is under selection at high elevation [18]. However, there are fundamental differences in embryonic growth rates [19] and Hb transcript abundance profiles [20,21] in early development between Galloanserae (ducks, chickens and geese) and other birds. Selection on Hb function at different stages of ontogeny may be a potent mechanism for improving hypoxia tolerance in birds. However, to understand its significance across the avian tree of life, this hypothesis must be evaluated in Neoaves, a large clade including over 95% of bird species [22].

To further our understanding of selection at high elevations over different ontogenetic stages, we tested for signatures of spatially varying selection in all Hb loci in a songbird, the hooded siskin (*Spinus magellanicus*). The hooded siskin occupies a broad elevational range in South America [23] and demonstrates little genetic structure across the west slope of the Peruvian Andes [24]. Natarajan *et al.* [17] identified a Hb *β*^A^ allele, present only in high-elevation individuals, that conferred an increase in Hb–O_2_ binding affinity compared to other alleles. This suggests that adult-expressed Hb isoforms may be under selection across elevation in hooded siskins. To test the role of *P*O_2_-related selection on Hb loci, including those expressed during embryonic development, we sequenced all seven Hb loci using a custom capture array and tested for signatures of spatially varying selection by comparing patterns of divergence in Hb loci to other loci sampled across the genome.

## 2. Methods

**Figure 1.**
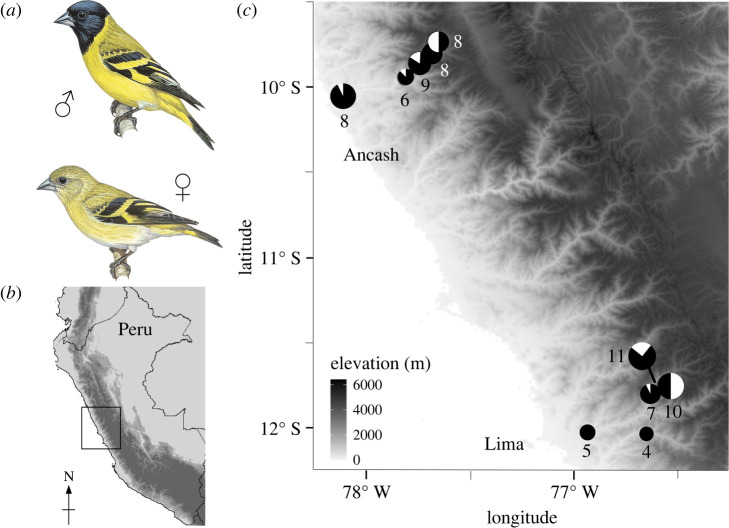
Peruvian hooded siskin sampling localities. (*a*) The hooded siskin; copyright © Lynx Edicions. (*b*) Peru; the study area in (*c*) is indicated with a black box. (*c*) Sample localities for Ancash and Lima transects. The number of individuals per locality is shown below each circle; coloration shows the proportion of *β*^H^135Val (black) and *β*^H^135Iso (white) alleles at each locality.

We sampled hooded siskins, *Spinus magellanicus,* across two 75 km long transects spanning approximately 4000 m of elevation in the Peruvian Andes (figure 1; electronic supplementary material, appendix). We sampled 37 and 39 post-fledge individuals in the departments of Lima and Ancash, respectively. On each transect, we acquired an average of eight individuals from the following elevations: 0–400 m; 700–1000 m; 1400–2300 m; 2500–3000 m; and above 3700 m. To provide context for the intraspecific results, we sampled another seven South American *Spinus* species, the distinct genetic lineage *S. magellanicus alleni*, and *S. notatus*, sister to all South American *Spinus* species [24,25] (electronic supplementary material, appendix).

We used a target-capture approach to sequence the seven complete Hb loci (*α*^π^, *α*^D^, *α*^A^; *β^ε^*, *β*^H^, *β*^A^, *β*^*ρ*^) based on Hb locus alignments constructed from nine-primaried oscines. We also targeted one or two exons from 363 randomly selected genes distributed across the autosomal genome (electronic supplementary material, file S1) based on the genome of a near-relative, the common canary *Serinus canaria* (GCA_000534875.1) [26]. We extracted DNA from muscle with the Qiagen DNeasy Blood and Tissue kit and prepared individual genomic libraries with the NEBNext Ultra DNA library prep kit and NEBNext Multiplex Oligos for Illumina. We aimed for an insert size of 400–500 bp, enriched the libraries using an MYbaits custom target enrichment kit, and pooled samples in equimolar proportions. We sequenced all individuals in one Illumina HiSeq 2500 lane (160 bp, paired-end) at the Keck Center at the University of Illinois, Champaign-Urbana. Raw reads were demultiplexed; we retained reads with a PHRED score >30.

We built locus-specific *pseudo*-references with aTRAM v. 1.04 [27] using Velvet v. 1.2.10 [28]. For paralogous genes like Hb loci, multiple sequences may be recovered in an aTRAM analysis. We initiated aTRAM runs with *Spinus* Hb cDNA sequences, then alternated between by-eye evaluation in Geneious v. 6 [29] and aTRAM to identify seven Hb loci. For each autosomal locus, we initiated aTRAM with the original probe and selected the longest contig as the reference. We aligned samples to each *pseudo*-reference with a sensitive search in Bowtie2 v. 2.2.6 [30], indexed reads in SAMtools v. 1.3.1 [31], and used Angsd v. 0.934 [32] to call variants. We filtered SNPs with VCFtools v. 0.1.16 [33] to exclude sites with less than 70% of individuals at a locality, transect-specific minor allele frequency under 0.05 and, for genome-wide loci, high linkage disequilibrium (LD ≥ 0.5). For interspecific data, we called SNPs using SAMtools and bcftools v. 1.3.1 [31].

We assessed population differentiation and selection among Peruvian hooded siskins. First, we conducted a principal component analysis using the genome-wide SNPs in R v. 4.1.0 [34]. Next, we calculated per-site F_ST_ [35] on each transect between individuals collected below 1000 m and those above 3700 m in VCFtools. To identify high-elevation population-specific allele frequency change on each transect, we calculated the per-site population branch statistic (PBS) [36]. We defined one focal high-elevation population, calculated pairwise *F*_ST_ among the focal population and the two low elevation populations (Lima < 1000 m; Ancash < 1000 m), then used a custom script (electronic supplementary material, file S2) in python v. 3.9.5 to compute the PBS. We estimated the total allele frequency change and the change observed from approximately 3000 m to over 3700 m on each transect. To assess significance, we asked if any non-synonymous sites in Hb loci demonstrated a value that was greater than 95% of the SNPs distributed across the autosome for *F*_ST_, PBS and total allele frequency change. Finally, we characterized the variation of non-synonymous Hb sites across the South American *Spinus* clade.

## 3. Results

We sequenced and aligned the complete coding sequences of all seven haemoglobin loci (electronic supplementary material, table S1). We recovered an average sequencing depth of 4.06× for Hb loci and 2.96× for genome-wide loci. We found high agreement between the *S. magellanicus* Hb genotypes inferred in this study and from published Sanger-sequenced cDNA (electronic supplementary material, file S3). After filtering, we recovered 249 variable loci from coding regions across the autosomal genome. Principal component analysis using the genome-wide loci revealed no structure within Peru in hooded siskins (electronic supplementary material, figure S1), concordant with previous work [24].

**Figure 2.**
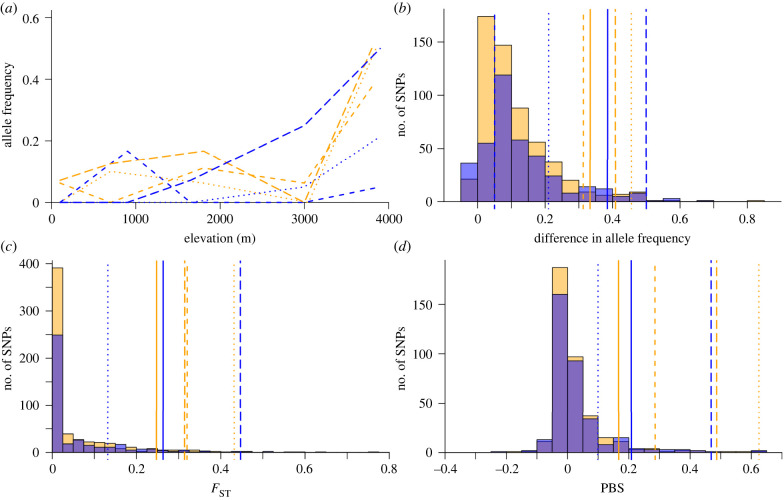
Haemoglobin non-synonymous candidate SNPs across elevation in hooded siskins*.* Results are coded by line type (*α*^π^60, short-dashed; *β*^A^21, dotted; *β*^H^135, long-dashed) and colour (blue, Lima; orange, Ancash); overlap of the transect-specific histograms appears purple. (*a*) Allele frequency across elevation. Statistics in (*b*–*d*) calculated between populations below 1000 m and above 3700 m; 95% threshold shown as a solid line. (*b*) Allele frequency differences. (*c*) F_ST_ (*d*) PBS. Close values in (*a*) and (*c*) slightly offset for visibility.

We tested for signatures of spatially varying selection by comparing the interrelated statistics of *F*_ST_, PBS and total allele frequency change at non-synonymous SNPs in Hb loci to SNPs from across the autosome. We identified three non-synonymous Hb sites in the top 5% of all SNPs genome-wide in at least one of these transect-specific statistics: embryonic Hb sites *α*^π^60 and *β*^H^135, and definitive Hb site *β*^A^21 (table 1; figure 2). All three Hb SNPs had a significant *F*_ST_ and PBS value in Ancash. In Lima, *β*^H^135 was the only significant outlier. For *β*^A^21, the high-elevation allele (*β*^A^21Thr) in Ancash was also at a higher frequency in the Lima high-elevation population, but the frequency difference was not statistically significant in Lima. We plotted allele frequency by elevation for *α*^π^60, *β*^A^21 and *β*^H^135, and discovered that a large proportion of the allele frequency change that occurred in Ancash was between the two highest sites (table 1; figure 2). By contrast, allele frequency in *β*^H^135 shifted as a linear function of elevation in Lima (adjusted *R*^2^ = 0.86, *p*-value 0.014).

**Table 1.** Non-synonymous haemoglobin candidate SNP variation in *Spinus magellanicus.* Values greater than 97% or 99% of genome-wide SNPs are indicated by * and **, respectively, and are in bold. O_2_ affinity summarized from [17]. Polarity change indicates a difference in polarity state (e.g. hydrophobic, polar, charged) between alternative amino acids.

	major allele < 1000 m	alternative allele	polarity change	change in O_2_ affinity	transect	allele frequency change	allele frequency change, 3000 to 3700 m	*F*_ST_	PBS
*α*^π^60	serine	phenylalanine	yes	—	Ancash	0.313	0.313	0.317*	0.286*
					Lima	0.05	0.05	—	—
*β*^A^21	alanine	threonine	yes	yes	Ancash	0.456*	0.5*	0.432*	0.625**
					Lima	0.21	0.16	0.132	0.099
*β*^H^135	valine	isoleucine	no	—	Ancash	0.409*	0.5*	0.316*	0.487**
					Lima	0.5*	0.25	0.447**	0.469**

We examined interspecific variation at these three non-synonymous Hb sites in South American *Spinus* (electronic supplementary material, figures S2, S3). For two sites, the highland allele we found in Ancash and Lima (*β*^A^21Thr; *β*^H^135Iso) only occurred in *Spinus* species with an upper range limit of at least 3000 m (table 2). Species that only occupy below 3000 m were fixed for the common allele at low elevation in hooded siskins (*β*^A^21Ala; *β*^H^135Val). In *S. magellanicus alleni*, a genetic lineage observed from lowland Argentina into mid-elevation Bolivia, highland alleles were only present in individuals sampled near 2500 m. We observed the variation at *α*^π^60 in hooded siskins from Peru alone; all other *Spinus* were fixed for *α*^π^60Ser.

**Table 2.** Variation of non-synonymous haemoglobin candidate SNPs across the *Spinus* clade. *N* indicates number of individuals. Alternative amino acids abbreviated as F: phenylalanine, T: threonine, I: isoleucine.

species	*N*	highest elevation (m)	*α*^π^60	*β*^A^21	*β*^H^135
*Spinus notatus*	1	≤ 3000	serine	alanine	valine
*S. cucullatus*	1	≤ 3000	S	A	V
*S. barbatus*	1	≤ 3000	S	A	V
*S. olivaceus*	1	≤ 3000	S	A	V
*S. siemiradzkii*	1	≤ 3000	S	A	V
*S. magellanicus alleni,* lowland	2	< 430	S	A	V
*S. magellanicus alleni,* Andes	1	∼ 2500	S	A/T	I
*S. magellanicus*, Peru	76	≥ 3000	S/F	A/T	V/I
*S. uropygialis*	1	≥ 3000	S	A/T	I
*S. crassirostris*	2	≥ 3000	S	T	I
*S. atratus*	1	≥ 3000	S	T	I

## 4. Discussion

We report the first evidence for selection on embryonic haemoglobin in high-elevation Neoaves. *β*^H^, which encodes the *β*-globin subunit of a major embryonic Hb isoform in passerines [21], contained the SNP *β*^H^135, that showed statistically significant population-specific differentiation on both transects. *α*^π^ encodes the *α* subunit of the embryonic Hb π; the variant *α*^π^60, which impacts amino acid side-chain polarity, demonstrated significant differentiation in Ancash, but not Lima. Last, a previously identified variant, the *β*^A^21 allele, which also changes amino acid polarity, is known to alter the O_2_ affinity of the definitive Hb isoforms HBD and HBA [17]. Here, we uncovered a striking signature of selection at *β*^A^21 in Ancash, and a suggestive trend in Lima. Together, these patterns suggest that the known functional effect at *β*^A^21 impacts whole-organism fitness. We conclude that hypoxic conditions likely exert selective pressure across the entire lifespan of this passerine, beginning in early development.

Avian embryonic haemoglobin isoforms contribute to O_2_ transport over a brief window, from days 3–6 of incubation [37]. Our results highlight that early development is a sensitive life stage with high mortality risk [38]. Further, performance (e.g. the ability to cope with hypoxia) over short, critical windows can contribute to lifetime success. An analogous scenario is the extreme hypoxia experienced by the bar-headed goose (*Anser indica*) during its single-day Himalayan migration [39]. These conclusions fit the intraspecific and interspecific patterns in *Spinus.* Embryonic *β*^H^135 and definitive *β*^A^21 exhibit local adaptation in Peruvian hooded siskins and segregate by elevation across South American *Spinus. Spinus* is a recent, rapidly diverged clade with widespread introgression [24]. A plausible explanation for the observed pattern is a single origin for each Hb allele and some combination of ancestral polymorphism, interspecific introgression and *P*O_2_-related selection at the species level.

Selection on embryonic Hb function may be a critical mechanism to improve oxygen transport in hypoxia in early avian development. Although deeply diverged from Neoaves, detailed studies in domestic fowl provide perspective on why this may be the case. In the chicken egg, oxygen in the first 6 days of incubation is severely limited due to the slow rate of gas diffusion across the aqueous inner membrane [4]. In the first week, the embryo requires little O_2_, and embryos with impaired Hb function may survive in normoxia [37]. However, in hypoxic conditions, lowland-ancestry embryo mortality is very high [40]. Many of the physiological mechanisms that allow embryos to tolerate hypoxia, like the synthesis of definitive Hb isoforms and their regulation through allosteric cofactors and changes to blood pH, develop at later ontogenetic stages [4]. Definitive Hb isoforms and their regulatory mechanisms develop earlier under hypoxic conditions [4,7,12,41]. However, oxygen transport from day 3–6 of incubation is unaltered by hypoxia exposure [37]; the growing embryo depends exclusively on embryonic Hb isoforms to transport diffused O_2_ to respiring tissues [7]. Thus, modifications that facilitate O_2_ binding and delivery for embryonic Hb likely contribute to improved embryo survivorship at low *P*O_2_. Studies that further examine the development of altricial passerines in hypoxic conditions will advance our mechanistic understanding of adaptation to hypoxia across the avian tree of life.

## 5. Conclusion

We report the first evidence of spatially varying selection associated with high elevation on Hb function at different stages of ontogeny in Neoaves. Our results dovetail with previous research that shows *P*O_2_-related selection on the primary sequence and relative isoform proportions of definitive Hb isoforms across the avian tree of life [15–17,42]. Embryonic Hb may be similarly predictable. In addition to our study, Graham *et al.* [18] showed significant differentiation between high and low lineages in embryonic Hb *β^ε^* in ducks (Galloanserae). We conclude that selection to improve hypoxia tolerance in avian embryos may be a critical, but understudied component to genetic adaptation to high elevation in birds.

## Data Availability

Sequence data are available at NCBI Sequence Read Archive as BioProject ID PRJNA857269. Specimen data are available in the electronic supplementary material, appendix. Electronic supplementary material information includes a description of the data as well as all the code used (see electronic supplementary material, file S2). A version of this manuscript in Spanish (Español) is available as electronic supplementary material, file S4. The data are provided in electronic supplementary material [43].
